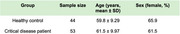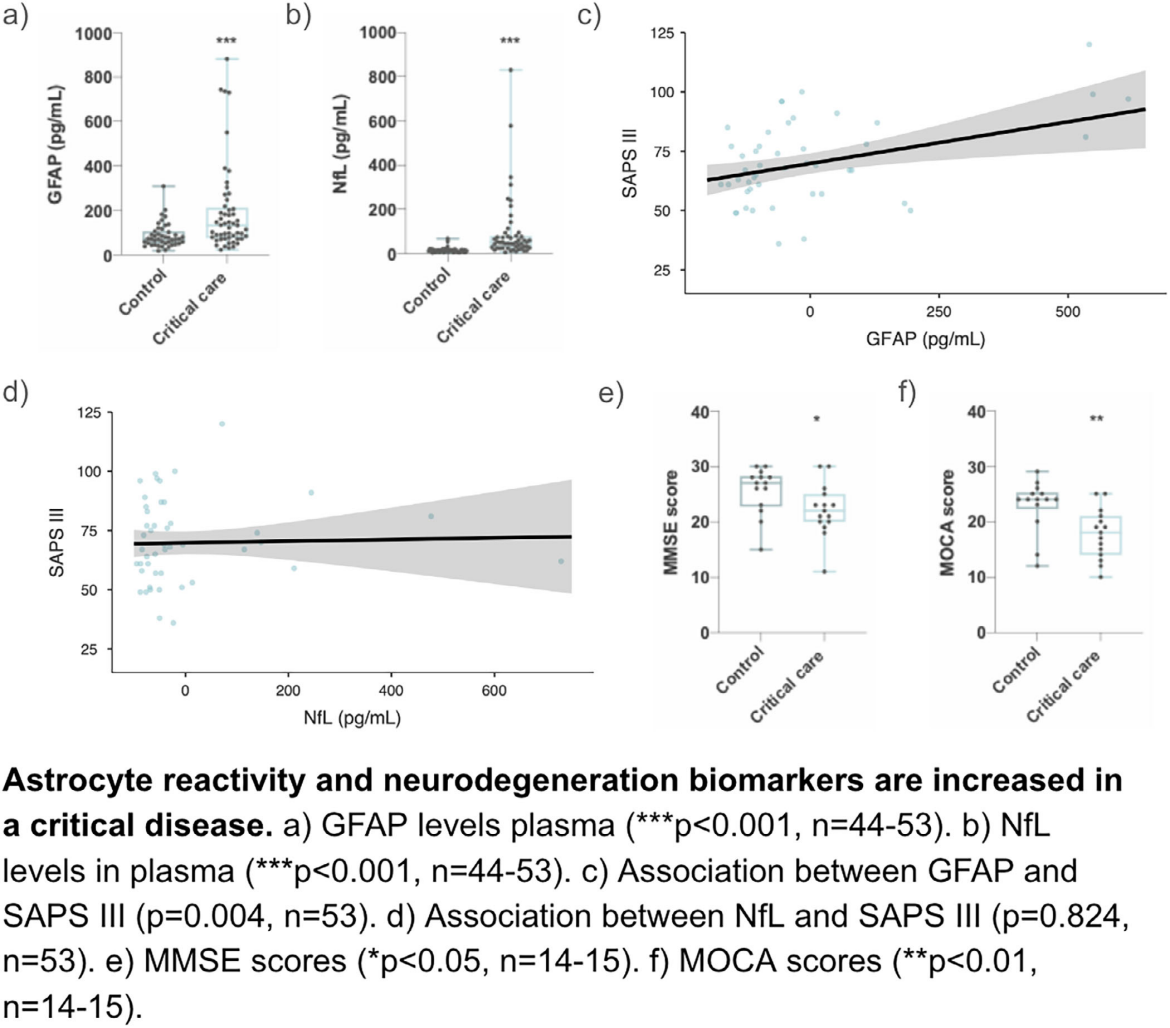# Astrocyte reactivity and neurodegeneration biomarkers in critical care patients and strategies for intervention

**DOI:** 10.1002/alz70857_103480

**Published:** 2025-12-24

**Authors:** Débora Guerini de Souza, Wyllians Vendramini Borelli, Fabiano Marcio Nagel, Marco Antônio De Bastiani, Christian Limberger, Isabela Scur Carrard, Pedro Rodrigues Vidor, João Pedro Uglione Da Ros, Lara Angi Souza, Monica Ochoa Nagel, Francieli Rohden, Eduardo R. Zimmer, Jaderson Costa da Costa, Diogo O. Souza

**Affiliations:** ^1^ Universidade Federal do Rio Grande do Sul, Porto Alegre, Rio Grande do Sul, Brazil; ^2^ Brain Institute of Rio Grande Do Sul, PUCRS, Porto Alegre, RS, Brazil; ^3^ Centro de Memória, Hospital Moinhos de Vento, Porto Alegre, RS, Brazil; ^4^ Hospital de Clínicas de Porto Alegre, Porto Alegre, Brazil; ^5^ Universidade Federal do Rio Grande do Sul, Porto Alegre, Brazil; ^6^ Universidade Luterana do Brazil, Canoas, RS, Brazil; ^7^ Brain Institute of Rio Grande do Sul (InsCer), PUCRS, Porto Alegre, Rio Grande do Sul, Brazil; ^8^ Instituto do Cérebro do Rio Grande do Sul, Porto Alegre, RS, Brazil

## Abstract

**Background:**

Individuals discharged from intensive care units (ICU) after recovery from severe disease are at high risk of developing neurodegeneration and long‐term cognitive impairment. Although common, this condition is poorly understood. Astrocyte reactivity, a heterogeneous response of astrocytes, is a common characteristic of neurodegenerative diseases. Here, we aimed to evaluate the impact of critical diseases on astrocyte reactivity, neurodegeneration, and cognition.

**Methods:**

Individuals from Hospital de Clínicas de Porto Alegre ICU, Brazil, above 40 years old were recruited. The Simplified Acute Physiology Score III (SAPS III) was used to estimate the mortality prediction of ICU patients. GFAP and NfL levels in plasma were measured with SIMOA to evaluate astrocyte reactivity and neuronal damage, respectively. Mini‐Mental State Examination (MMSE) and the Montreal Cognitive Assessment (MOCA) were used for the cognitive screening. Statistical analyses were conducted with ANCOVA and generalized linear models, accounting for age and sex, with a significant threshold of *p* <0.05.

**Results:**

We included 44 healthy controls and 53 individuals admitted to ICU due to critical diseases (demographics are shown in Table 1). The critical disease group presented higher levels of GFAP (Figure 1a, F 15.094, *p* <0.001) and NfL (Figure 1b, F 12.32, *p* <0.001) compared with healthy controls. GFAP levels were positively associated with SAPS III score (Figure 1c, β=0.0351, 95%CI 0.011, 0.0588, *p* = 0.004), while NfL levels were not (Figure 1d, β=0.00347, 95%CI ‐0.027, 0.0339, *p* = 0.824). MMSE (Figure 1e, F=5.371, *p* = 0.029) and MOCA (Figure 1f, F=11.75, *p* = 0.002) scores showed a significant difference between groups; however, both tests did not associate with GFAP and NfL levels.

**Conclusions:**

Critical care diseases influence astrocyte reactivity and neurodegeneration, suggesting that severe systemic diseases may be associated with neuropathological processes. Besides, astrocyte reactivity was associated with mortality prediction scores. These results emphasize the need for further research into GFAP and NfL as biomarkers to better understand and manage neurodegeneration in critical care settings.